# Reference Values of Right Ventricular Volumes and Ejection Fraction by Three-Dimensional Echocardiography in Adults: A Systematic Review and Meta-Analysis

**DOI:** 10.3389/fcvm.2021.709863

**Published:** 2021-09-23

**Authors:** Shitong Wang, Shuyu Wang, Qing Zhu, Yonghuai Wang, Guangyuan Li, Fanxin Kong, Jun Yang, Chunyan Ma

**Affiliations:** Department of Cardiovascular Ultrasound, First Affiliated Hospital of China Medical University, Shenyang, China

**Keywords:** three-dimensional echocardiography, reference value, right ventricle, volume, ejection fraction

## Abstract

**Objective:** This study was conducted in order to determine the reference values for right ventricular (RV) volumes and ejection fraction (EF) using three-dimensional echocardiography (3DE) and to identify sources of variance through a systematic review and meta-analysis.

**Methods:** This systematic review was preregistered with the International Prospective Register of Systematic Reviews (https://www.crd.york.ac.uk/PROSPERO/) (CRD42020211002). Relevant studies were identified by searches of the PubMed, Embase, and Cochrane Library databases through October 12, 2020. Pooled reference values were calculated using the random-effects model weighted by inverse variance. Meta-regression analysis and Egger's test were used to determine the source of heterogeneity. A subgroup analysis was performed to evaluate the reference values across different conditions.

**Results:** The search identified 25 studies of 2,165 subjects. The mean reference values were as follows: RV end-diastolic volume, 100.71 ml [95% confidence interval (CI), 90.92–110.51 ml); RV end-systolic volume, 44.19 ml (95% CI, 39.05–49.33 ml); RV end-diastolic volume indexed, 57.01 ml/m^2^ (95% CI, 51.93–62.08 ml/m^2^); RV end-systolic volume indexed, 25.41 ml/m^2^ (95% CI, 22.58–28.24 ml/m^2^); and RVEF, 56.20% (95% CI, 54.59–57.82%). The sex- and age-specific reference values were assessed according to the studies reporting the values of different sexes and age distributions, respectively. In addition, the vendor- and software-specific reference values were analyzed. The meta-regression analysis revealed that sex, frame rate, pulmonary artery systolic pressure, and software packages were associated with variations in RV volumes (*P* < 0.05). Inter-vendor and inter-software discrepancies may explain the variability of RVEF.

**Conclusions:** The reference values for RV volumes and RVEF using 3DE were assessed. The confounders that impacted the variability in RV volumes or RVEF contained the sex, frame rate, pulmonary artery systolic pressure, inter-vendor discrepancies, and inter-software discrepancies.

## Introduction

Right ventricular (RV) function is involved in the diagnosis and prognosis of many cardiac disorders such as congenital and acquired heart diseases (1, 2). However, because of the RV complex anatomy and functional characteristics, the application of conventional two-dimensional echocardiography (2DE) for quantifying its function in the clinical setting is limited (1–3). Similar to the left ventricle, the assessment of RV volumes and RV ejection fraction (EF) can quantitatively reflect RV systolic function. Recently, with the development of three-dimensional echocardiography (3DE), current guidelines have recommended that 3DE can be applied to accurately assess RV volumes and RVEF (4, 5).

However, the lack of large population-based reference values for RV volumes and RVEF has precluded their clinical use. Most previous studies have analyzed RV volumes and RVEF in healthy populations from a single center with relatively small sample sizes. Meanwhile, the determination of universal reference values for RV volumes and RVEF by 3DE through multicenter large-sample studies should be required to enable standardized clinical application of 3DE for quantitatively assessing RV function. Thus, the major objectives of this study are to (1) assess the reference values for RV volumes and RVEF by 3DE in healthy adults and (2) identify the potential confounders that may contribute to the variability in published reference values for RV volumes and RVEF as assessed by 3DE.

## Methods

### Search Protocol

The systematic review and meta-analysis was performed in accordance with the Preferred Reporting Items for Systematic Reviews and Meta-Analyses (PRISMA) guidelines (6). The PubMed, Embase, and Cochrane Library databases were systematically searched for articles published through October 12, 2020. The search strategy is shown in Supplementary Material 1. The search was limited to human studies published in English. Moreover, studies lacking complete published articles or that were published as conference abstracts, reviews, and editorial comments were excluded from the analysis. This systematic review was prospectively registered with the International Prospective Register of Systematic Reviews (https://www.crd.york.ac.uk/PROSPERO/) on November 2, 2020 (CRD42020211002).

### Selection Criteria

We selected studies that reported RV volumes and/or RVEF using 3DE in “healthy” or “normal” adults, had a sample size of more than 30, and were published in the English language. Criteria for “healthy” or “normal” adults were as follows: age ≥ 18 years; no history or symptoms of cardiovascular or lung disease; no use of medication; no systemic disease; no obesity; no pregnancy; no cardiovascular risk factors, that is, arterial hypertension, diabetes, or dyslipidemia; and normal ECG and physical examination findings. Moreover, we excluded studies involving control subjects with cardiovascular risk factors and those lacking adequate description of the baseline characteristics of their control populations.

### Study Selection

Two investigators (STW and SYW) independently performed the title screening, removal of duplicates, abstract reviews, and full-text reviews based on predefined selection criteria to identify relevant articles. Furthermore, the references of included articles were manually screened for additional eligible studies. Disagreements were resolved by consensus or discussion with a third investigator (CM).

### Data Collation

The following data were extracted and entered into an electronic database: (1) study: first author and year of publication; (2) demographic characteristics: numbers of control subjects, age, sex proportion, and country; (3) clinical parameters: systolic blood pressure (SBP), diastolic blood pressure (DBP), heart rate (HR), body surface area (BSA), and body mass index (BMI); (4) echocardiographic methodological parameters: frame rate (FR), vendors, and software packages; and (5) echocardiographic parameters: pulmonary artery systolic pressure (PASP), RV end-diastolic volume (EDV), RV end-systolic volume (ESV), RV EDV indexed by BSA (EDVi), RV ESV indexed by BSA (ESVi), and RVEF. If multiple articles were published using the same dataset, the study with the largest sample was assessed.

### Quality Assessment

To evaluate the quality of the included studies, we selected 12 items relevant to this systematic review and meta-analysis based on the quality assessment methodology and similar systematic reviews and meta-analyses (7, 8).

### Statistical Analysis

All statistical analyses were performed using Stata 15 (StataCorp LLC, 2017). The mean and 95% confidence interval (CI) of the RV volumes and RVEF were calculated to create pooled estimates using random-effects models weighted by inverse variance. Inter-study statistical heterogeneity was assessed by the Cochrane *Q* statistic and quantified by the *I*^2^ statistic. The results are presented as forest plots, the standard method to illustrate the results of individual studies and overall meta-analyses. We performed a meta-regression analysis to assess whether any demographic, clinical, or echocardiographic methodological parameters influenced the variability of RV volumes or RVEF in the “normal” adults; values of *P* < 0.05 were considered significant. In addition, a subgroup analysis was performed to determine reference values in the specific conditions. A quantitative evaluation of publication bias was performed using the funnel plot and Egger's test; values of *P* < 0.1 indicated significant bias. If publication bias was significant, the “trim and fill” method was used to examine whether our estimates were changed after regulating the missing studies (9).

## Results

### Study Selection

**Figure 1:**
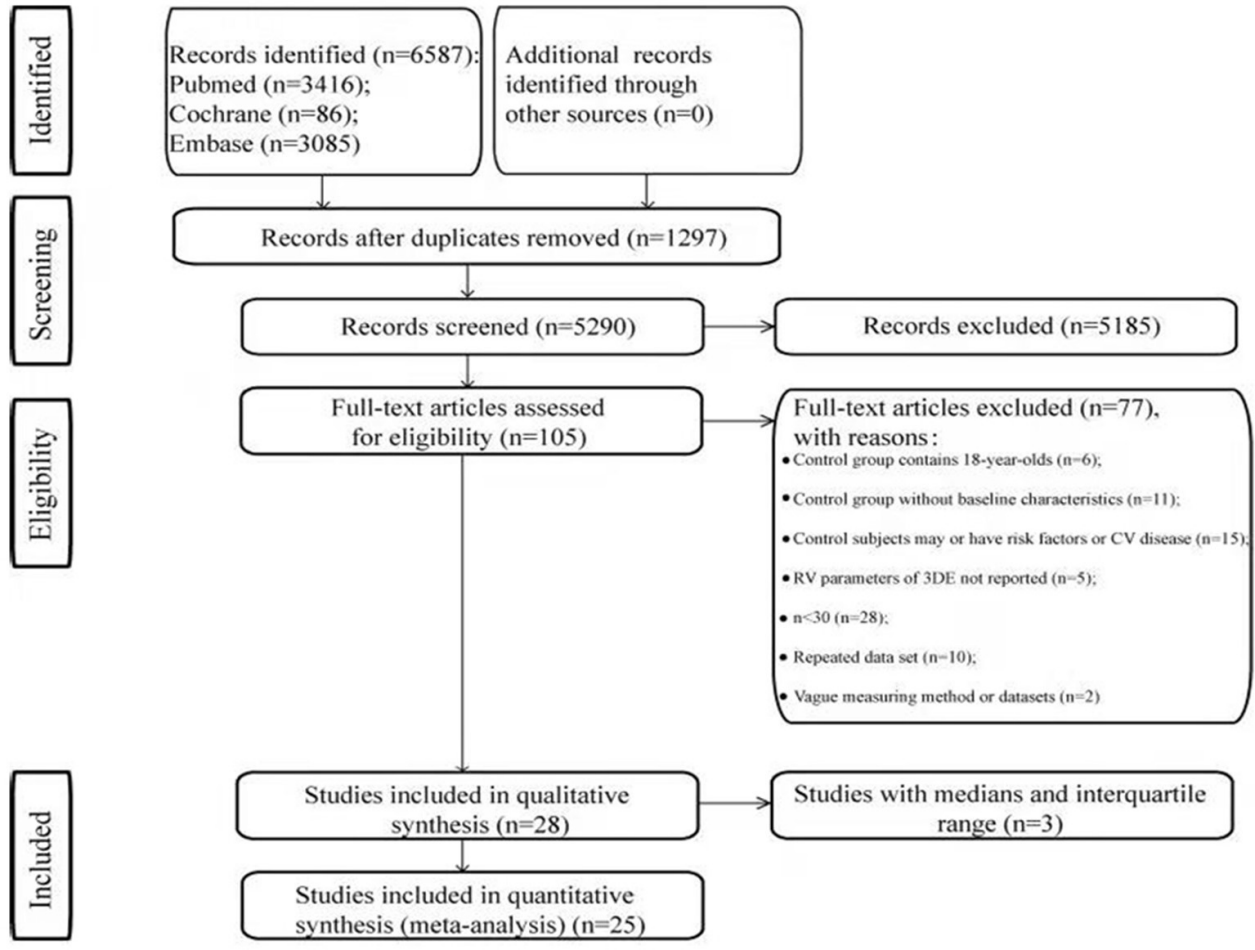
Flowchart of the study selection process.

A total of 6,587 articles were identified through a systematic search of three electronic databases. After the exclusion of duplicates and triplicates (*n* = 1,297), 5,290 articles were screened for relevance. In the title and abstract review process, 5,185 articles were excluded. Thereafter, 105 articles were subjected to full-text review, during which process 77 articles were excluded. A total of 28 articles met our inclusion criteria (Figure 1). Of them, nine studies had recruited only “normal” adults, whereas the remaining studies had recruited “normal” adults as the control population. Three studies did not report the mean RV volumes and RVEF (10–12). Finally, 25 studies with 30 datasets were included in this meta-analysis (13–37). Among the included 25 studies, two studies had repeated datasets but used different parameters to perform the meta-analysis (26, 36).

### Summary of Selected Studies

All 25 studies of 2,165 patients were eligible for the meta-analysis. The baseline characteristics of the included studies are listed in Table 1. The mean age ranged from 23 to 67 years, and men accounted for 6-100% of the study population. Philips, GE, and Toshiba Medical System 3DE products were used to capture RV imaging. TomTec, EchoPAC, 3D QLAB, and Toshiba 3DT speckle tracking software packages were used to perform the analyses.

**Table 1:** The baseline characteristics of the included studies.

**References**	**Number**	**Gender, male, %**	**Age^**a**^, years**	**BMI^**a**^, kg/m^**2**^**	**BSA^a^**	**SBP^**a**^**, **mmHga**	**DBP^**a**^**, **mmHga**	**HR^**a**^**, **bpma**	**PASP^**a**^**, **mmHg**	**Vendor**	**FR^**a**^**, **frames/s^**a**^**	**Software**
Buonauro et al. (14)	30	63	67 ± 9	25 ± 3	NR	128 ± 13	79 ± 9	68 ± 11	26.7 ± 4.6	GE (Vivid E9)	>25	TomTec
Buonauro et al. (18)	50	10	42 ± 9	23 ± 6	NR	122 ± 13	75 ± 10	74 ± 10	24.4 ± 5.0	GE (Vivid E95)	>25	TomTec
Sun et al. (28)	30	47	54 ± 13	NR	1.8 ± 0.2	120 ± 9	76 ± 5	69 ± 10	29.6 ± 2.9	GE (Vivid E9)	45 ± 6	TomTec
Clemmensen et al. (15)	41	59	51 ± 2	24 ±2	NR	NR	NR	NR	NR	GE (Vivid 9)	>25	TomTec
Lv et al. (25)	46	74	45 ± 13	NR	1.7 ± 0.2	117 ± 7	76 ± 8	68 ± 11	NR	Philips (EPIQ 7C)	19–23	TomTec
Smith et al. (27)	60	40	41 ± 12	NR	NR	NR	NR	NR	25.9 ± 4.3	Toshiba Medical System (Artida)	13.7 ± 0.8	Toshiba 3DT speckle tracking software
Tadic et al. (29)	58	53	48 ± 9	25 ± 3	1.9 ± 0.2	123 ± 6	75 ± 5	NR	23.0 ± 5.0	GE (Vivid 7)	20–30	EchoPAC
Tadic et al. (30)	35	6	53 ± 8	25 ± 3	1.7 ± 0.2	128 ± 8	73 ± 7	NR	23.0 ± 8.0	GE (Vivid 7)	20–30	EchoPAC
van der Zwaan et al. (32) Vitarelli et al. (33)	41	56	27 ± 8	22 ± 3	1.9 ± 0.2	121 ± 14	73 ± 8	64 ± 13	NR	Philips (IE33)	14–38	TomTec
	35	100	28 ± 11	22 ± 2	1.9 ± 0.2	121 ± 7	75 ± 6	70 ± 9	21.6 ± 6.2	GE (Vivid E9)	NR	EchoPAC
Vitarelli et al. (34)	30	43	54 ± 15	23 ± 3	1.9 ± 0.2	114 ± 15	63 ± 8	65 ± 8	22.0 ± 3.0	GE (Vivid E9)	NR	TomTec
Lakatos et al. (23)	300	50	45 ± 16	24 ± 4	1.8 ± 0.2	132 ± 15	77 ± 11	69 ± 14	25.3 ± 5.7	Philips (EPIQ 7) and GE (Vivid E95)	NR	TomTec
Addetia et al. (13)	245	56	42 ± 12	NR	1.8 ± 0.2	NR	NR	NR	NR	Philips (IE33) and GE (Vivid E9)	27 ± 7	TomTec
Aune et al. (37)	166	48	29-79	25 ± 3	1.9 ± 0.2	NR	NR	NR	NR	Philips (IE33)	NR	3D QLAB
Gopal et al. (20)	71	49	56 ± 14	NR	NR	NR	NR	NR	NR	Philips	15–18	TomTec
Kjaergaard et al. (21)	54	48	59 ± 14	25 ± 4	1.9 ± 0.2	136 ± 18	82 ± 10	67 ± 8	NR	Philips (Sonos 7500)	NR	TomTec
McGhieb et al. (26)^b^	147	50	45 ± 14	24 ± 3	1.9 ± 0.2	127 ± 15	80 ± 9	62 ± 11	NR	Philip (IE33and EPIQ 7)	27 ± 8	TomTec
Tamborini et al. (31)	245	49	48 ± 17	NR	1.8 ± 0.2	NR	NR	NR	26.0 ± 4.0	Philips (IE33)	32 ± 3	TomTec
van Grootelb et al. (36)^b^	147	50	45 ± 14	24 ± 3	1.9 ± 0.2	127 ± 15	80 ± 9	62 ± 11	NR	Philips (IE33 and EPIQ 7)	NR	TomTec
D'Andrea et al. (16)	250	58	28 ± 10	NR	1.8 ± 0.6	121 ± 7	76 ± 4	73 ± 11	17.5 ± 4.6	GE (Vivid E9)	16–24	TomTec
D'Andrea et al. (17)	80	63	57 ± 5	NR	1.9 ± 0.1	130 ± 12	75 ± 12	72 ± 11	18.7 ± 8.1	GE (Vivid E9)	16–24	TomTec
Lai et al. (22)	48	54	23 ± 5	NR	NR	NR	NR	NR	NR	GE (Vivid 7)	NR	TomTec
Lakatos et al. (24)	30	36	50 ± 13	24 ± 3	1.8 ± 0.2	124 ± 13	75 ± 8	66 ± 10	16.1 ± 5.4	Philips (EPIQ 7G)	NR	TomTec
Esposito et al. (19)	43	NR	29 ± 6	23 ± 3	NR	119 ± 12	71 ± 9	69 ± 11	NR	GE (Vivid E9)	NR	TomTec
Vitarelli et al. (35)	30	63	46 ± 13	26 ± 4	1.8 ± 0.2	120 ± 8	74 ± 6	69 ± 9	22.0 ± 3.0	GE (Vivid E9)	NR	TomTec
Maffessanic et al. (10)^c^	507	49	45 ± 16	23 ± 3	1.8 ± 0.2	121 ± 15	73 ± 10	68 ± 11	24.5 ± 5.4	GE (Vivid E9) and Philips (IE33)	26–40	TomTec
Zhang et al. (11)^c^	40	19	60 ± 7	22	1.7 ± 0.1	125	81	77	NR	Philips (IE33)	NR	3D QLAB
Moceric et al. (12)^c^	55	48	33.0	NR	NR	NR	NR	68 ± 12	NR	Philips (IE33 and EPIQ-7)	24–28	TomTec

*BMI, body mass index; BSA, body surface area; DBP, diastolic blood pressure; FR, frame rate; HR, heart rate; PASP, pulmonary artery systolic pressure; NR, not reported; SBP, systolic blood pressure*.

a *Data are presented as mean ± standard deviation or median (range)*.

b *Studies included repeated databases*.

c *Studies that met the inclusion criteria but were not included in the meta-analysis*.

### The Reference Values

The mean RVEF evaluated by 3DE was reported in all 30 datasets. The included datasets with RV volumes and RVEF are presented in Table 2.

**Table 2:** Summary of meta-analysis results of RV volumes by 3DE.

**Parameters**	**Datasets**	**Numbers**	**Mean**	**95% CI**	***I* ^**2**^**	**Cochrane *Q***	***P***
EDV	23	1,610	100.71	90.92-110.51	98.6	1,588	<0.001
ESV	22	1,575	44.19	39.05-49.33	98.4	1,338	<0.001
EDVi	19	1,475	57.01	51.93-62.08	98.5	1,238	<0.001
ESVi	17	1,410	25.41	22.58-28.24	98.3	964	<0.001
RVEF	30	2,165	56.20	54.59-57.82	97.7	1,281	<0.001

*The significance level was set at P < 0.05. A random-effects model was used to assess inter-study heterogeneity*.

*3DE, three-dimensional echocardiography; CI, confidence interval; EDV, end-diastolic volume; EDVi, end-diastolic volume indexed by body surface area; ESV, end-systolic volume; ESVi, end-systolic volume indexed by body surface area; RV, right ventricular; RVEF, RV ejection fraction*.

**Figure 2:**
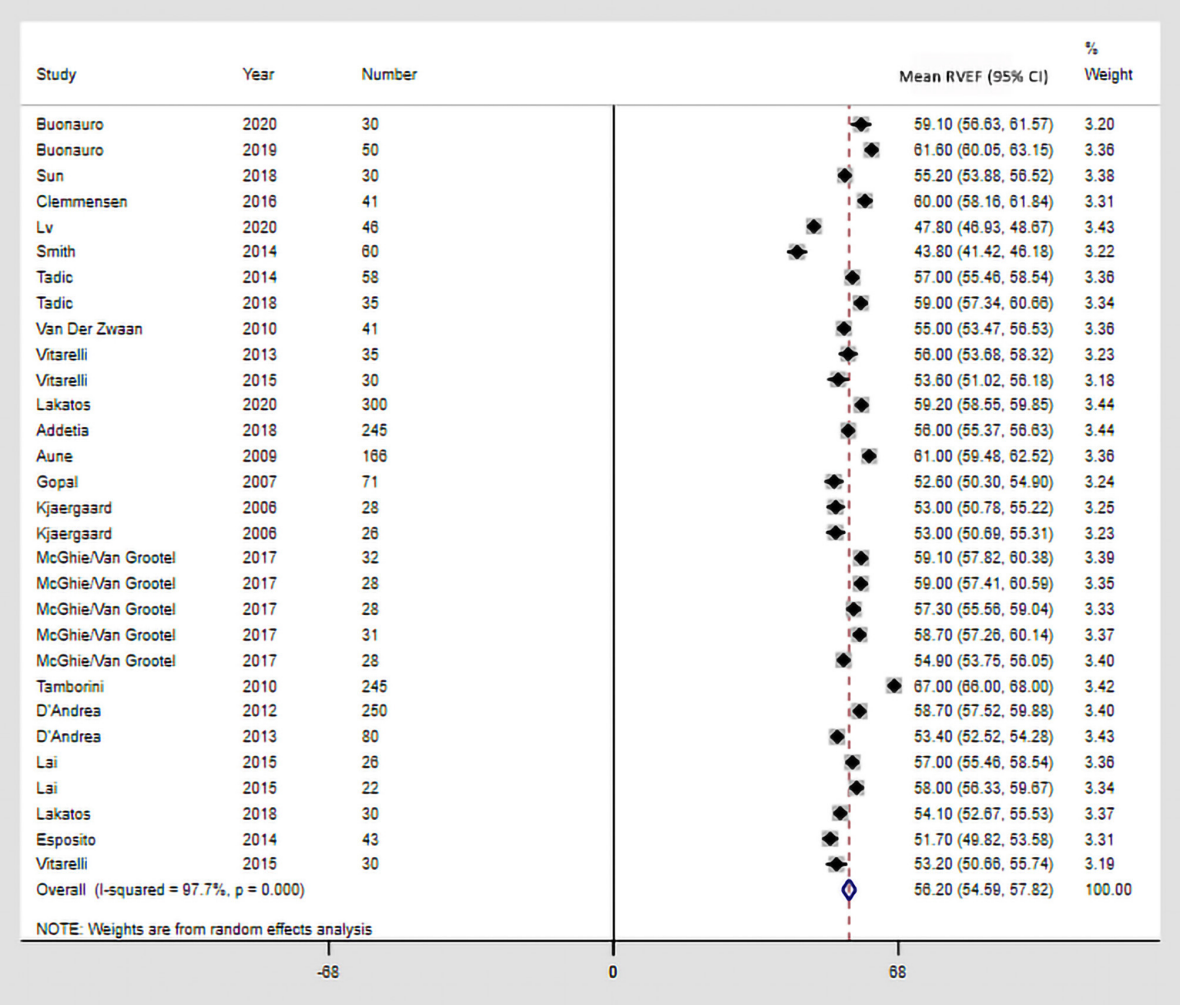
Reference values of RVEF by 3DE. The square represents the mean of the point effect estimate of each study. The square size indicates the weight of the study. The horizontal line extending from either side of the square represents the 95% CI. The diamond reflects the pooled overall consequence. 3DE, three-dimensional echocardiography; CI, confidence interval; RVEF, right ventricular ejection fraction.

The EDV ranged from 56.50 to 130.00 ml (mean, 100.71 ml; 95% CI, 90.92–110.51 ml), and the ESV ranged from 21.80 to 63.90 ml (mean, 44.19 ml; 95% CI, 39.05–49.33 ml) (Supplementary Figures 1A,B). The EDVi ranged from 40.00 to 73.50 ml/m^2^ and the ESVi ranged from 16.00 to 33.40 ml/m^2^. The mean and 95% CI EDVi and ESVi are presented in Supplementary Figures 2A,B. The Cochrane *Q* statistic and *I*^2^ inconsistency indicated the presence of inter-study heterogeneity for RV volumes. The mean RVEF was 56.20% (95% CI, 54.59–57.82%; *I*^2^ = 97.7%), with a Cochrane *Q* statistic of 1,281 (*P* < 0.001; Figure 2). The reference values for RV volumes and RVEF stratified by different vendors and software packages are presented in Table 3.

**Table 3:** Vendor- and software-specific reference values for RV volumes and RVEF by 3DE.

	**Vendor**			**Software packages**			
**RV parameters**	**GE**	**Philips**	**Toshiba Medical System**	**TomTec**	**EchoPAC**	**3D QLAB**	**Toshiba 3DT speckle tracking software**
EDV (ml)	93.38 (76.32-110.43)	105.82 (95.60-116.03)	NR	103.85 (92.19-115.51)	89.42 (73.82-105.02)	77.00 (73.50-80.50)	NR
ESV (ml)	41.62 (33.27-49.98)	45.50 (39.08-51.92)	NR	45.43 (39.34-51.52)	40.04 (32.20-47.88)	30.00 (28.17-31.83)	NR
EDVi (ml/m^2^)	56.75 (42.82-70.68)	57.40 (51.99-62.81)	NR	59.31 (54.24-64.37)	47.48 (32.78-62.18)	40.00 (38.33-41.67)	NR
ESVi (ml/m^2^)	27.88 (22.53-33.22)	24.89 (21.52-28.26)	NR	26.16 (23.11-29.20)	24.00 (22.20-25.80)	16.00 (15.09-16.91)	NR
RVEF (%)	56.71 (55.14-58.28)	56.36 (52.93-59.79)	43.80 (41.42 to 46.18)	56.35 (54.60-58.10)	57.45 (55.77-59.12)	61.00 (59.48-62.52)	43.80 (41.42-46.18)

*Data are expressed as mean (95% CI). Abbreviations as in Table 2*.

Ten studies reported the overall sex-specific RV volumes and/or RVEF (13, 20–23, 26, 31, 32, 36, 37). Five studies reported the age-specific RV volumes and/or RVEF using similar age groups (23, 26, 31, 36, 37). The sex- and age-specific reference RV volumes and RVEF are presented in Table 4.

**Table 4:** Sex- and age-specific reference values of RV volumes and RVEF by 3DE.

	**EDV (ml)**	**ESV (ml)**	**EDVi (ml/m^**2**^)**	**ESVi (ml/m^**2**^)**	**RVEF (%)**
**Gender**					
Male	119.44 (97.50-141.39)	51.87 (36.77-66.98)	61.60 (54.95-68.24)	27.01 (22.56-31.46)	56.46 (54.38-58.53)
Female	92.22 (74.92-109.52)	38.05 (26.95-49.15)	54.11 (48.43-59.80)	22.02 (18.60-25.44)	59.03 (56.30-61.76)
Overall	105.86 (91.21-120.51)	44.94 (35.70-54.18)	57.85 (53.46-62.24)	24.52 (21.75-27.29)	57.73 (55.98-59.48)
**Age, years**					
<30	99.68 (83.37-115.99)	38.53 (27.36-49.70)	56.40 (52.44-60.37)	21.91 (18.07-25.75)	61.23 (57.87-64.58)
30–39	92.62 (82.53-102.72)	35.47 (27.92-43.03)	50.97 (46.54-55.40)	19.56 (16.28-22.85)	61.36 (58.75-63.98)
40–49	90.46 (76.10-104.83)	34.80 (25.24-44.36)	48.37 (42.53-54.20)	18.77 (15.18-22.37)	61.77 (57.74-65.81)
50–59	89.33 (67.30-111.35)	34.75 (24.01-45.49)	49.26 (40.65-57.88)	19.46 (15.32-23.59)	60.55 (57.08-64.03)
≥60	87.19 (72.09-102.30)	33.49 (22.84-44.15)	48.62 (41.56-55.68)	19.02 (14.35-23.70)	61.57 (56.32-66.81)
Overall	90.92 (84.31-97.54)	35.02 (31.07-38.97)	50.33 (47.45-53.20)	19.59 (17.93-21.26)	61.29 (59.71-62.86)

*Data are expressed as mean (95% CI). Abbreviations as in Table 2*.

### Sources of Variability

We independently analyzed age, sex, HR, SBP, DBP, BMI, BSA, PASP, FR, vendor, and software packages using meta-regression analysis to determine whether any influenced the variability of RV volumes and RVEF (Supplementary Table 1). The variability of ESV was impacted by sex and PASP (*P* < 0.05). The discrepancy between software packages impacted the variability of EDVi (*P* = 0.044). Additionally, FR was also associated with the bias of RV volumes (*P* < 0.05) but not bias of RVEF. The variation of RVEF was mainly correlated with the inter-vendor and inter-software discrepancies (*P* < 0.05). Noticeably, when the study used the Toshiba Medical System and the Toshiba 3DT speckle tracking software was omitted, the impact of vendor and software packages to RVEF was not significant [*P*_(vendor)_ = 0.839; *P*_(software)_ = 0.473], but the influence of FR did not change (*P* = 0.995). Other parameters showed no significant impact on variability of RV volumes and RVEF.

### Publication Bias

**Figure 3:**
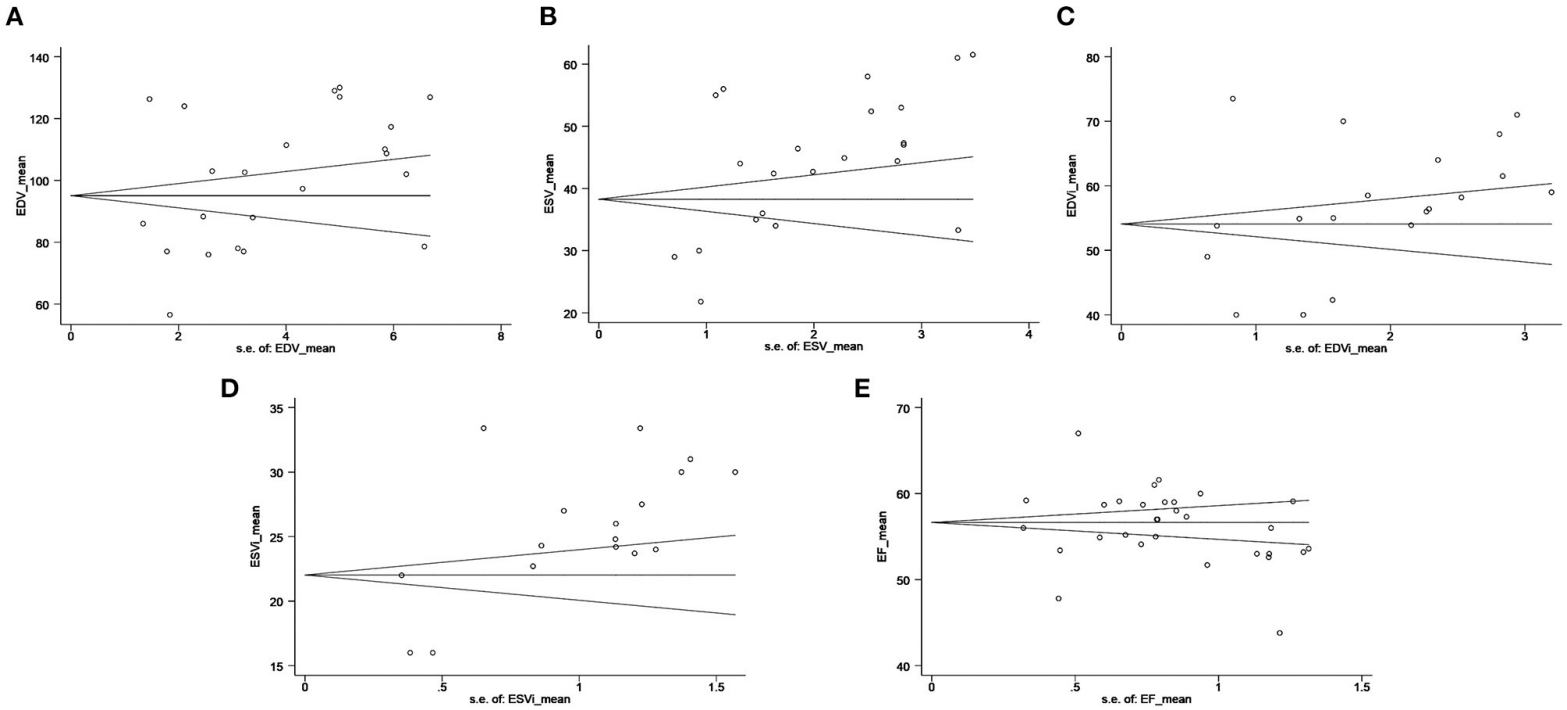
Funnel plot for RV volumes and RVEF: **(A)** RV EDV (*P* = 0.402), **(B)** RV ESV (*P* = 0.011), **(C)** RV EDVi (*P* = 0.357), **(D)** RV ESVi (*P* = 0.007), and **(E)** RVEF (*P* = 0.669). The standard error of the effect estimate is plotted on the horizontal axis. The mean is plotted on the vertical axis. The circle presents each included study. The black lines indicate the pooled mean and 95% CI. 3DE, three-dimensional echocardiography; CI, confidence interval; EDV, end-diastolic volume; EDVi, EDV indexed by body surface area; ESV, end-systolic volume; ESVi, ESV indexed by body surface area; RV, right ventricular; RVEF, RV ejection fraction.

Publication bias was not found for EDV, EDVi, or RVEF; however, publication bias was noted for ESV and ESVi (Figure 3). Moreover, an additional analysis using the “trim and fill” method for ESV and ESVi suggested that hypothetically missing studies did not substantially change the estimates (Supplementary Figure 3).

### Quality Assessment

All studies included in our meta-analysis were appraised using the quality checklist items (Supplementary Table 2). All studies described the echocardiographic image acquisition and postprocessing protocols. A reproducibility analysis was performed in 19 studies.

## Discussion

This study analyzed the reference values for RV volumes and RVEF using 3DE in “normal” adults and assessed the impact of the potential confounders on the variation in the reported values of RV volumes and RVEF.

The RV, viewed as the “forgotten ventricle,” has gained wide attention in the past few years. Increasing evidence has revealed that RV function can play a crucial role in determining the functional status and prognosis of patients with various cardiovascular diseases such as ischemic and non-ischemic cardiomyopathy, pulmonary arterial hypertension, congenital heart disease, and heart failure (38–40). In common clinical practice, RV function is assessed by 2DE parameters such as tricuspid annular systolic excursion and fractional area change (5). However, its asymmetrical and complex crescent shape and retrosternal location make it difficult to visualize the entire RV chamber in a two-dimensional view; thus, the accuracy and reproducibility of 2DE for RV function assessments are limited (41). Hence, other multiparametric approaches should be used to more comprehensively and accurately evaluate RV function, particularly in complex clinical case (3). Cardiac magnetic resonance (CMR), the gold standard for RV function assessment, can precisely and quantitatively evaluate RV volumes and RVEF to reflect the global RV systolic function and load conditions. However, in actual clinical practice, CMR cannot be used to analyze the RV function of all patients because of its high cost, various contraindications, and non-portability (3). Hence, an alternative non-invasive method to evaluate RV volumes and RVEF is needed.

The American Society of Echocardiography, European Association of Cardiovascular Imaging, and British Society of Echocardiography recently recommended the use of 3DE for quantifying the RV chamber and performing functional analyses (4, 5). Compared with conventional 2DE, 3DE can provide more precise anatomical definitions of the RV without requiring geometrical assumptions; thus, it is able to overcome the inherent limitations of 2DE (2). Studies reported that 3DE-derived RV volumes and RVEF could strongly correlate with CMR imaging findings, even if RV volumes may be underestimated by 3DE vs. CMR (42–45). RV volumes and RVEF by 3DE have been considered parameters for the diagnosis and outcomes of cardiopulmonary diseases or RV possible subclinical changes in patient workups (12, 15, 18, 46, 47). The RVEF <45% can reflect RV systolic dysfunction universally, but reference values for RV volumes and RVEF by 3DE are insufficiently investigated (4).

The advent of 3DE has provided novel insight into RV functional assessments. The universal reference values are critical for the implementation of RV volumes and RVEF by 3DE since the clinical parameters for ensuring more standard evaluations of clinical changes in RV function vary across a broad range of physiological and pathological conditions in adults (4, 5). However, most studies reporting RV volumes and RVEF have been single-center studies with small sample sizes. Lakatos et al. evaluated the reference values for RV volumes and RVEF on 3DE in 300 European and Japanese individuals; however, studies with larger populations with boarder age groups and multiethnic expansion were required to strengthen their findings (23). The findings of another multicenter study conducted at three tertiary centers in Italy may be limited by race and ethnicity homogeneity (10). The updated recommendation in 2015 reported the reference values for RV volume and RVEF on 3DE by pooling the data of 15 studies. However, larger-sample studies are needed to more accurately define these reference values (5). Although our systematic review and meta-analysis extended the reference values for RV volumes and expanded the sample size for RVEF, further investigations are still required.

We evaluated the data of 2,165 individuals from 25 studies and analyzed the reference values for RV volumes and RVEF using 3DE. The EDV, ESV, EDVi, and ESVi were reported by 76, 72, 56, and 48% of the eligible studies, respectively. Data from different studies were combined in a meta-analytical format, and relevant representative estimates of the reference values for RV volumes and RVEF by 3DE were provided.

The sex- and age-specific reference values for RV volumes and RVEF using 3DE were analyzed in our study. Previous studies demonstrated that the male population had lower RVEF and higher RV volumes than the female population (10, 20, 21, 26, 31). The potential reason for this may be the differences in biometric characteristics between women and men (10). Meanwhile, the RV volumes and RVEF were also probably correlated with age distribution. Thus, we analyzed the age-specific reference values despite the lack of a significant correlation. In most studies, RVEF tended to gradually decrease with age (23, 26, 36). However, Maffessanti et al. reported that RVEF was positively correlated with age (10). The potential reason for this difference was that the RVEF did not increase until old age (>70 years) (36). Thus, more relative studies should be performed with wider age distributions to specify the correlations and validate the accuracy of these age-specific reference values.

The variability in references values of RV volumes and RVEF by 3DE identified by the current study is probably due to differences in populations as well as echocardiographic vendors and analytical software (48). The European Association of Cardiovascular Imaging stated that even if the inter-vendor and inter-software variability of advanced echocardiographic parameters are recognized, a similar variability of some standard echocardiographic parameters cannot be excluded *a priori* (49). The variations between vendors may result from differences in analytical algorithms for image formatting, interpolation techniques, and numeric filters (50). The difference between analytical software packages may also affect the RV volumes and RVEF due to the use of different internal analysis algorithms. TomTec Imaging System as a vendor-independent software has been widely applied, while 3D QLAB, EchoPAC, and Toshiba 3DT software packages are also used to assess RV volumes and RVEF. Based on the above, vendor- and software-specific reference values were necessary for the analysis. Thus, this study assessed the vendor- and software-specific reference values. However, the 3D QLAB, EchoPAC, and Toshiba 3DT software packages were used by only one or two studies each. Further investigations should be performed to validate their reliability.

### Source of Bias

Our meta-regression analysis indicated that age, BSA, BMI, HR, SBP, and DBP failed to explain the source of bias in our meta-analysis, but other studies showed a weak statistical correlation with RVEF (23). Thus, we must be cautious when hypothesizing that these features do not impact RV volumes and RVEF. The study findings indicated that the variability of ESV was likely to be explained by sex and PASP. PASP was the pressure load for right ventricle. A previous study demonstrated that the increase in RV contractility might be a compensatory mechanism attempting to cope with the increased pressure load (11). The FR of more than 20–25 frames/s is usually required (5). Insufficient temporal resolution may lead to errors in the identification of end diastole and systole, resulting in the underestimation of EDV and overestimation of ESV (44). However, in the study of Smith et al., FR was markedly lower than the values of other studies. The authors stated that when applying to the Toshiba Medical System and Toshiba software, the low FR may not hide the pattern of contraction (27). FR may not be as important for their study, so it cannot be controlled effectively. Our finding demonstrated that the FR may explain the variability of RV volumes despite exclusion of the study with the lower FR. The discrepancy among the GE, Philips, and Toshiba Medical System products may explain the RVEF variability. However, the reported values of RVEF for the Toshiba Medical System were markedly lower than those for the GE and Philips products. When the relevant study was omitted, our result demonstrated that the discrepancy between GE and Philips may not explain the variability of RVEF. Similarly, the discrepancy of TomTec, EchoPAC, 3D QLAB, and Toshiba 3DT speckle tracking software may explain the variability of RVEF. However, when the study that used the Toshiba 3DT speckle tracking software was ignored, the variability of RVEF was not explained by the software packages. Therefore, the Toshiba Medical System and Toshiba 3DT speckle tracking software may be confounders leading to RVEF bias. Meanwhile, the discrepancy noted of the TomTec, EchoPAC, and 3D QLAB products was likely to explain the EDVi variability.

### Limitations

The current study has some limitations. First, similar to other meta-analyses reporting the reference values, remarkable heterogeneity is an innate limitation although we attempted to explore the source of heterogeneity and define reference values in various conditions (8). Among the included studies, only eight aimed to evaluate the reference values to enroll healthy populations. This may have contributed to the limited accuracy of our findings. Meanwhile, the imbalanced distribution of vendors and software packages limited the accuracy of those values. In particular, only one study used the Toshiba Medical System and Toshiba 3DT speckle tracking software, which might further limit the comparability of our values. Thus, the vendor- and software-specific reference values may not necessarily be applicable to other studies and require further validation. Few studies provided FR; thus, further studies are required to validate the influence on reference values. Moreover, software versions may also be an important confounder that impacts RV volumes and RVEF variations due to the wide enrollment period and the continuous software updates. However, half of the included studies did not specify the software versions used and each offered version was used by just one or two studies, which limits the further analysis of software versions. In the future, more studies can elucidate the influence of software versions.

## Conclusion

This study assessed the reference values for RV volumes and RVEF using 3DE in “normal” adults, and the sex, frame rate, pulmonary artery systolic pressure, and inter-vendor and inter-software discrepancies impacted the variability in RV volumes or RVEF. This assessment of reference values for RV volumes and RVEF using 3DE is expected to play a role in improving the clinical application.

## Data Availability Statement

The original contributions presented in the study are included in the article/Supplementary Material, further inquiries can be directed to the corresponding author/s.

## Author Contributions

CM and ShiW were involved in the conception and design of the study. ShiW and ShuW collected the data. ShiW, YW, QZ, GL, FK, and CM analyzed and interpreted the data and performed the meta-analysis. ShiW, JY, and CM prepared and revised the manuscript. All authors have approved the final manuscript.

## Funding

This work was supported by a general program from the National Natural Science Foundation of China (Grant No. 81871373), “Xingliao Talents Plan” Project (Grant No. XLYC1905001), and Shenyang Science and Technology Bureau (Grant No. 20-205-4-014).

## Conflict of Interest

The authors declare that the research was conducted in the absence of any commercial or financial relationships that could be construed as a potential conflict of interest.

## Publisher's Note

All claims expressed in this article are solely those of the authors and do not necessarily represent those of their affiliated organizations, or those of the publisher, the editors and the reviewers. Any product that may be evaluated in this article, or claim that may be made by its manufacturer, is not guaranteed or endorsed by the publisher.

## Supplementary Material

Supplementary Material 1 The search strategy.

Supplementary Table 1 Meta-regression results for RV volumes and RVEF by 3DE.

Supplementary Table 2 Quality assessment of included studies.

Supplementary Figure 1 The reference values for EDV and ESV by 3DE.

Supplementary Figure 2 The reference values for EDVi and ESVi by 3DE.

Supplementary Figure 3 Funnel plots with ESV and ESVi estimates using the “trim and fill” method.

## References

[1] Kossaify A. Echocardiographic assessment of the right ventricle, from the conventional approach to speckle tracking and three-dimensional imaging, and insights into the “Right Way” to explore the forgotten chamber. Clin Med Insights Cardiol. (2015) 9:65–75. 10.4137/CMC.S27462 26244034 PMC4493918

[2] Seo Y Ishizu T Ieda M Ohte N. Right ventricular three-dimensional echocardiography: the current status and future perspectives. J Echocardiogry. (2020) 18:149–59. 10.1007/s12574-020-00468-8 32239383

[3] Addetia K Muraru D Badano LP Lang RM. New directions in right ventricular assessment using 3-dimensional echocardiography. JAMA Cardiol. (2019) 4:936–44. 10.1001/jamacardio.2019.2424 31339508

[4] Zaidi A Knight DS Augustine DX Harkness A Oxborough D Pearce K . Echocardiographic assessment of the right heart in adults: a practical guideline from the British Society of Echocardiography. Echo Res Pract. (2020) 7:G19–41. 10.1530/ERP-19-0051 32105053 PMC7077526

[5] Lang RM Badano LP Mor-Avi V Afilalo J Armstrong A Ernande L . Recommendations for cardiac chamber quantification by echocardiography in adults: an update from the American Society of Echocardiography and the European Association of Cardiovascular Imaging. Eur Heart J Cardiovasc Imaging. (2015) 16:233–70. 10.1093/ehjci/jev014 25712077

[6] Moher D Liberati A Tetzlaff J Altman D. Preferred reporting items for systematic reviews and meta-analyses: the PRISMA statement. PLoS Med. (2009) 6:e1000097. 10.1371/journal.pmed.1000097 19621072 PMC2707599

[7] Downs S Black N. The feasibility of creating a checklist for the assessment of the methodological quality both of randomised and non-randomised studies of health care interventions. J Epidemiol Commun Health. (1998) 52:377–84. 10.1136/jech.52.6.377 9764259 PMC1756728

[8] Levy PT Machefsky A Sanchez AA Patel MD Rogal S Fowler S . Reference ranges of left ventricular strain measures by two-dimensional speckle-tracking echocardiography in children: a systematic review and meta-analysis. J Am Soc Echocardiogr. (2016) 29:209–25 e6. 10.1016/j.echo.2015.11.016 26747685 PMC4779733

[9] Duval S Tweedie R. Trim and fill: a simple funnel-plot-based method of testing and adjusting for publication bias in meta-analysis. Biometrics. (2000) 56:455–63. 10.1111/j.0006-341X.2000.00455.x 10877304

[10] Maffessanti F Muraru D Esposito R Gripari P Ermacora D Santoro C . Age-, body size-, and sex-specific reference values for right ventricular volumes and ejection fraction by three-dimensional echocardiography: a multicenter echocardiographic study in 507 healthy volunteers. Circ Cardiovasc Imaging. (2013) 6:700–10. 10.1161/CIRCIMAGING.113.000706 23811752

[11] Zhang L Zhang P Qi H Wang Z Luo T Lu M . Right ventricular function in pulmonary hypertension due to left heart disease by two-dimensional speckle tracking and real time three-dimensional echocardiography. Acta Cardiol. (2016) 71:473–82. 10.1080/AC.71.4.3159702 27594364

[12] Moceri P Duchateau N Gillon S Jaunay L Baudouy D Squara F . Three-dimensional right ventricular shape and strain in congenital heart disease patients with right ventricular chronic volume loading. Eur Heart J Cardiovasc Imaging. (2020) jeaa189. 10.1093/ehjci/jeaa189. [Epub ahead of print]. 32756985

[13] Addetia K Maffessanti F Muraru D Singh A Surkova E Mor-Avi V . Morphologic analysis of the normal right ventricle using three-dimensional echocardiography-derived curvature indices. J Am Soc Echocardiogr. (2018) 31:614–23. 10.1016/j.echo.2017.12.009 29402505 PMC5936650

[14] Buonauro A Santoro C Galderisi M Canora A Sorrentino R Esposito R . Impaired right and left ventricular longitudinal function in patients with fibrotic interstitial lung diseases. J Clin Med. (2020) 9:587. 10.3390/jcm9020587 32098133 PMC7073641

[15] Clemmensen TS Eiskjaer H Logstrup BB Andersen MJ Mellemkjaer S Poulsen SH. Echocardiographic assessment of right heart function in heart transplant recipients and the relation to exercise hemodynamics. Transpl Int. (2016) 29:909–20. 10.1111/tri.12793 27159372

[16] D'Andrea A Riegler L Morra S Scarafile R Salerno G Cocchia R . Right ventricular morphology and function in top-level athletes: a three-dimensional echocardiographic study. J Am Soc Echocardiogr. (2012) 25:1268–76. 10.1016/j.echo.2012.07.020 22898244

[17] D'Andrea A Riegler L Nunziata L Scarafile R Gravino R Salerno G . Right heart morphology and function in heart transplantation recipients. J Cardiovasc Med. (2013) 14:648–58. 10.2459/JCM.0b013e32835ec634 23442808

[18] Buonauro A Sorrentino R Esposito R Nappi L Lobasso A Santoro C . Three-dimensional echocardiographic evaluation of the right ventricle in patients with uncomplicated systemic lupus erythematosus. Lupus. (2019) 28:538–44. 10.1177/0961203319833786 30885082

[19] Esposito R Galderisi M Schiano-Lomoriello V Santoro A De Palma D Ippolito R . Nonsymmetric myocardial contribution to supranormal right ventricular function in the athlete's heart: combined assessment by speckle tracking and real time three-dimensional echocardiography. Echocardiography. (2014) 31:996–1004. 10.1111/echo.12499 24373023

[20] Gopal AS Chukwu EO Iwuchukwu CJ Katz AS Toole RS Schapiro W . Normal values of right ventricular size and function by real-time 3-dimensional echocardiography: comparison with cardiac magnetic resonance imaging. J Am Soc Echocardiogr. (2007) 20:445–55. 10.1016/j.echo.2006.10.027 17484982

[21] Kjaergaard J Sogaard P Hassager C. Quantitative echocardiographic analysis of the right ventricle in healthy individuals. J Am Soc Echocardiogr. (2006) 19:1365–72. 10.1016/j.echo.2006.05.012 17098140

[22] Lai CT Wong SJ Ip JJ Wong WK Tsang KC Lam WW . Plasma levels of high sensitivity cardiac troponin T in adults with repaired tetralogy of fallot. Sci Rep. (2015) 5:14050. 10.1038/srep14050 26360613 PMC4566090

[23] Lakatos BK Nabeshima Y Tokodi M Nagata Y Tosér Z Otani K . Importance of nonlongitudinal motion components in right ventricular function: three-dimensional echocardiographic study in healthy volunteers. J Am Soc Echocardiogr. (2020) 33:995–1005.e1. 10.1016/j.echo.2020.04.002 32620323

[24] Lakatos BK Tokodi M Assabiny A Toser Z Kosztin A Doronina A . Dominance of free wall radial motion in global right ventricular function of heart transplant recipients. Clin Transplant. (2018) 32:e13192. 10.1111/ctr.13192 29315873

[25] Lv Q Sun W Wang J Wu C Li H Shen X . Evaluation of biventricular functions in transplanted hearts using 3-dimensional speckle-tracking echocardiography. J Am Heart Assoc. (2020) 9:e015742. 10.1161/JAHA.119.015742 32370590 PMC7660853

[26] McGhie JS Menting ME Vletter WB Frowijn R Roos-Hesselink JW van der Zwaan HB . Quantitative assessment of the entire right ventricle from one acoustic window: an attractive approach. Eur Heart J Cardiovasc Imaging. (2017) 18:754–62. 10.1093/ehjci/jew165 27502293

[27] Smith BC Dobson G Dawson D Charalampopoulos A Grapsa J Nihoyannopoulos P. Three-dimensional speckle tracking of the right ventricle: toward optimal quantification of right ventricular dysfunction in pulmonary hypertension. J Am Coll Cardiol. (2014) 64:41–51. 10.1016/j.jacc.2014.01.084 24998127

[28] Sun M Cao X Guo Y Tan X Dong L Pan C . Long-term impacts of hemodialysis on the right ventricle: assessment *via* 3-dimensional speckle-tracking echocardiography. Clin Cardiol. (2018) 41:87–95. 10.1002/clc.22857 29363796 PMC6490018

[29] Tadic M Cuspidi C Pencic B Sljivic A Ivanovic B Neskovic A . High-normal blood pressure impacts the right heart mechanics: a three-dimensional echocardiography and two-dimensional speckle tracking imaging study. Blood Press Monit. (2014) 19:145–52. 10.1097/MBP.0000000000000043 24695214

[30] Tadic M Zlatanovic M Cuspidi C Stevanovic A Celic V Damjanov N . Systemic sclerosis impacts right heart and cardiac autonomic nervous system. J Clin Ultrasound. (2018) 46:188–94. 10.1002/jcu.22552 29064088

[31] Tamborini G Marsan NA Gripari P Maffessanti F Brusoni D Muratori M . Reference values for right ventricular volumes and ejection fraction with real-time three-dimensional echocardiography: evaluation in a large series of normal subjects. J Am Soc Echocardiogr. (2010) 23:109–15. 10.1016/j.echo.2009.11.026 20152691

[32] van der Zwaan HB Helbing WA Boersma E Geleijnse ML McGhie JS Soliman OI . Usefulness of real-time three-dimensional echocardiography to identify right ventricular dysfunction in patients with congenital heart disease. Am J Cardiol. (2010) 106:843–50. 10.1016/j.amjcard.2010.05.008 20816126

[33] Vitarelli A Capotosto L Placanica G Caranci F Pergolini M Zardo F . Comprehensive assessment of biventricular function and aortic stiffness in athletes with different forms of training by three-dimensional echocardiography and strain imaging. Eur Heart J Cardiovasc Imaging. (2013) 14:1010–20. 10.1093/ehjci/jes298 23299399

[34] Vitarelli A Mangieri E Terzano C Gaudio C Salsano F Rosato E . Three-dimensional echocardiography and 2D-3D speckle-tracking imaging in chronic pulmonary hypertension: diagnostic accuracy in detecting hemodynamic signs of right ventricular (RV) failure. J Am Heart Assoc. (2015) 4:e001584. 10.1161/JAHA.114.001584 25792128 PMC4392438

[35] Vitarelli A Terzano C Saponara M Gaudio C Mangieri E Capotosto L . Assessment of right ventricular function in obstructive sleep apnea syndrome and effects of continuous positive airway pressure therapy: a pilot study. Can J Cardiol. (2015) 31:823–31. 10.1016/j.cjca.2015.01.029 25980631

[36] van Grootel RWJ Menting ME McGhie J Roos-Hesselink JW van den Bosch AE. Echocardiographic chamber quantification in a healthy Dutch population. Neth Heart J. (2017) 25:682–90. 10.1007/s12471-017-1035-7 29019026 PMC5691816

[37] Aune E Baekkevar M Rodevand O Otterstad JE. The limited usefulness of real-time 3-dimensional echocardiography in obtaining normal reference ranges for right ventricular volumes. Cardiovasc Ultrasound. (2009) 7:35. 10.1186/1476-7120-7-35 19580673 PMC2713207

[38] Sanders JL Koestenberger M Rosenkranz S Maron BA. Right ventricular dysfunction and long-term risk of death. Cardiovasc Diagn Ther. (2020) 10:1646–58. 10.21037/cdt-20-450 33224778 PMC7666957

[39] Santens B Van De Bruaene A De Meester P D'Alto M Reddy S Bernstein D . Diagnosis and treatment of right ventricular dysfunction in congenital heart disease. Cardiovasc Diagn Ther. (2020) 10:1625–45. 10.21037/cdt-20-370 33224777 PMC7666946

[40] Sanz J Sanchez-Quintana D Bossone E Bogaard HJ Naeije R. Anatomy, function, and dysfunction of the right ventricle: JACC state-of-the-art review. J Am Coll Cardiol. (2019) 73:1463–82. 10.1016/j.jacc.2018.12.076 30922478

[41] Tanabe K. Three-dimensional echocardiography- role in clinical practice and future directions. Circ J. (2020) 84:1047–54. 10.1253/circj.CJ-20-0239 32404540

[42] van der Zwaan HB Geleijnse ML McGhie JS Boersma E Helbing WA Meijboom FJ . Right ventricular quantification in clinical practice: two-dimensional vs. three-dimensional echocardiography compared with cardiac magnetic resonance imaging. Eur J Echocardiogr. (2011) 12:656–64. 10.1093/ejechocard/jer107 21810828

[43] Myhr KA Kristensen CB Pedersen FHG Hassager C Vejlstrup N Mattu R . Accuracy and sensitivity of three-dimensional echocardiography to detect changes in right ventricular volumes: comparison study with cardiac magnetic resonance. Int J Cardiovasc Imaging. (2020) 37:493–502. 10.1007/s10554-020-02017-x 32914403

[44] Shimada YJ Shiota M Siegel RJ Shiota T. Accuracy of right ventricular volumes and function determined by three-dimensional echocardiography in comparison with magnetic resonance imaging: a meta-analysis study. J Am Soc Echocardiogr. (2010) 23:943–53. 10.1016/j.echo.2010.06.029 20797527

[45] Knight DS Grasso AE Quail MA Muthurangu V Taylor AM Toumpanakis C . Accuracy and reproducibility of right ventricular quantification in patients with pressure and volume overload using single-beat three-dimensional echocardiography. J Am Soc Echocardiogr. (2015) 28:363–74. 10.1016/j.echo.2014.10.012 25499839 PMC4346278

[46] Ryo K Goda A Onishi T Delgado-Montero A Tayal B Champion HC . Characterization of right ventricular remodeling in pulmonary hypertension associated with patient outcomes by 3-dimensional wall motion tracking echocardiography. Circ Cardiovasc Imaging. (2015) 8:e003176. 10.1161/CIRCIMAGING.114.003176 26038432

[47] Tadic M Celic V Cuspidi C Ilic S Zivanovic V Marjanovic T. How does subclinical hyperthyroidism affect right heart function and mechanics? J Ultrasound Med. (2016) 35:287–95. 10.7863/ultra.15.03054 26715657

[48] Zhang X Zhu H Tian X Zhu L Luo S Yuan J. three-dimensional echocardiography-derived strain values acquired by a novel analysis program. Echocardiography. (2018) 35:1841–6. 10.1111/echo.14143 30255620

[49] Galderisi M Cosyns B Edvardsen T Cardim N Delgado V Di Salvo G . Standardization of adult transthoracic echocardiography reporting in agreement with recent chamber quantification, diastolic function, and heart valve disease recommendations: an expert consensus document of the European Association of Cardiovascular Imaging. Eur Heart J Cardiovasc Imaging. (2017) 18:1301–10. 10.1093/ehjci/jex244 29045589

[50] Muraru D Cucchini U Mihaila S Miglioranza MH Aruta P Cavalli G . Left ventricular myocardial strain by three-dimensional speckle-tracking echocardiography in healthy subjects: reference values and analysis of their physiologic and technical determinants. J Am Soc Echocardiogr. (2014) 27:858–71 e1. 10.1016/j.echo.2014.05.010 24975996

