# Supplementary material for: Reference Values of Right Ventricular Volumes and Ejection Fraction by Three-Dimensional Echocardiography in Adults: A Systematic Review and Meta-Analysis
Source: Front Cardiovasc Med. 2021 Sep 23;8:709863. doi: 10.3389/fcvm.2021.709863 (PMC8495027; doi:10.3389/fcvm.2021.709863)
Supplement: Supplementary Material 1 — The search strategy. [file Data_Sheet_1.doc]

**Supplementary Material 1. The search strategy**

**Pubmed (3416)**

("heart ventricles"[All fields] OR "right ventricle"[All fields] OR "right ventricles"[All fields] OR "right ventricular"[All fields] OR "RV"[All fields]) AND (("3D"[All fields] OR "three dimension"[All fields] OR "three dimensional"[All fields] OR "three-dimension"[All fields] OR "three-dimensional"[All fields]) AND ("echocardiography"[All fields] OR "imaging"[All fields] OR "transthoracic echocardiography"[All fields] OR "TTE"[ALL fields] OR "3DE"[All fields] OR "echocardiogram"[All fields] OR "echocardiographic"[All fields] OR “ultrasound”[All fields]))

**Cochrane(86)**

#1 ("Heart Ventricles"):ti,ab,kw OR ("Right Ventricles"):ti,ab,kw OR ("Right Ventricle"):ti,ab,kw OR ("Right ventricular"):ti,ab,kw OR ("RV"):ti,ab,kw

#2 ("3D"):ti,ab,kw OR ("three dimension"):ti,ab,kw OR ("three dimensional"):ti,ab,kw OR ("three-dimension"):ti,ab,kw OR ("three-dimensional"):ti,ab,kw

#3 ("echocardiography"):ti,ab,kw OR ("imaging"):ti,ab,kw OR ("transthoracic echocardiography"):ti,ab,kw OR ("TTE"):ti,ab,kw OR ("3DE"):ti,ab,kw

#4 ("echocardiographic"):ti,ab,kw OR ("echocardiograom"):ti,ab,kw OR (“ultrasound”):ti,ab,kw

#5 #3 OR #4

#6 #1 AND #2 AND #5

**Embase (3085)**

#1 'heart ventricles' OR 'right ventricles' OR 'right ventricle' OR 'right ventricular' OR 'rv'

#2 '3d' OR 'three dimension' OR 'three dimensional' OR 'three-dimension' OR 'three-dimensional'

#3 'echocardiography' OR 'imaging' OR 'transthoracic echocardiography' OR 'tte' OR '3de' OR 'echocardiographic' OR 'echocardiograom' OR 'ultrasound'

#4 #1 AND #2 AND #3 AND ([article]/lim OR [article in press]/lim) AND [humans]/lim
