# Supplementary material for: Reference Values of Right Ventricular Volumes and Ejection Fraction by Three-Dimensional Echocardiography in Adults: A Systematic Review and Meta-Analysis
Source: Front Cardiovasc Med. 2021 Sep 23;8:709863. doi: 10.3389/fcvm.2021.709863 (PMC8495027; doi:10.3389/fcvm.2021.709863)
Supplement: Supplementary Table 1 — Meta-regression results for RV volumes and RVEF by 3DE. [file Table_1.docx]

**Supplementary Table 1**. Meta-regression results for RV volumes and RVEF by 3DE

| Confounders |  | *P* value | | | | | | | | |
| --- | --- | --- | --- | --- | --- | --- | --- | --- | --- | --- |
|  |  | EDV |  | ESV |  | EDVi |  | ESVi |  | EF |
| Age |  | 0.405 |  | 0.270 |  | 0.536 |  | 0.772 |  | 0.675 |
| Gender |  | 0.238 |  | **0.034** |  | 0.772 |  | 0.268 |  | 0.387 |
| BMI |  | 0.819 |  | 0.898 |  | 0.648 |  | 0.779 |  | 0.861 |
| BSA |  | 0.450 |  | 0.230 |  | NA |  | NA |  | 0.926 |
| HR |  | 0.129 |  | 0.413 |  | 0.799 |  | 0.105 |  | 0.784 |
| SBP |  | 0.209 |  | 0.251 |  | 0.400 |  | 0.941 |  | 0.582 |
| DBP |  | 0.138 |  | 0.247 |  | 0.539 |  | 0.866 |  | 0.108 |
| PASP |  | 0.072 |  | **0.010** |  | 0.171 |  | 0.137 |  | 0.641 |
| FR |  | **0.007** |  | **0.003** |  | **0.005** |  | **0.005** |  | 0.141 |
| Vendor |  | 0.168 |  | 0.425 |  | 0.888 |  | 0.334 |  | 0.018 |
| Software |  | 0.301 |  | 0.340 |  | **0.044** |  | 0.146 |  | 0.019 |

*The significance level was set at P<0.05.*

*BMI, body mass index; BSA, body surface area; SBP, systolic blood pressure; HR,heart rate; PASP, pulmonary artery systolic presseure; FR, frame rate; 3DE, three-dimensional echocardiography; CI, confidence interval; EDV, end-diastolic volume; EDVi, EDV indexed by BSA; ESV, end-systolic volume; ESVi, ESV indexed by BSA; RV, right ventricular; RVEF, RV ejection fraction.*
