# Supplementary material for: Reference Values of Right Ventricular Volumes and Ejection Fraction by Three-Dimensional Echocardiography in Adults: A Systematic Review and Meta-Analysis
Source: Front Cardiovasc Med. 2021 Sep 23;8:709863. doi: 10.3389/fcvm.2021.709863 (PMC8495027; doi:10.3389/fcvm.2021.709863)
Supplement: Supplementary Table 2 — Quality assessment of included studies. [file Table_2.docx]

**Supplementary Table 2**. Quality assessment of included studies

| Author |  | Year |  | objective defined |  | Outcome described |  | Charachteristics described |  | Confounders described |  | Main findings outlined |  | Heterogenous population |  | Imaging protocol |  | Individuals generating data blinded to outcomes |  | Sonographers blinded  to outcomes |  | Was reproducibility  Analysis performed? |  | Case/controls recruited over same time periods |
| --- | --- | --- | --- | --- | --- | --- | --- | --- | --- | --- | --- | --- | --- | --- | --- | --- | --- | --- | --- | --- | --- | --- | --- | --- |
| Buonauro |  | 2020 |  | Y |  | Y |  | Y |  | Y |  | Y |  | N |  | Y |  | NS |  | NS |  | Y |  | Y |
| Buonauro |  | 2019 |  | Y |  | Y |  | Y |  | Y |  | Y |  | N |  | Y |  | NS |  | NS |  | N |  | Y |
| Sun |  | 2018 |  | Y |  | Y |  | Y |  | Y |  | Y |  | N |  | Y |  | N |  | N |  | Y |  | Y |
| Clemmensen |  | 2016 |  | Y |  | Y |  | Y |  | Y |  | Y |  | N |  | Y |  | Y |  | Y |  | N |  | Y |
| Lv |  | 2020 |  | Y |  | Y |  | Y |  | Y |  | Y |  | Y |  | Y |  | Y |  | NS |  | Y |  | Y |
| Smith |  | 2013 |  | Y |  | Y |  | Y |  | Y |  | Y |  | N |  | Y |  | NS |  | NS |  | Y |  | Y |
| Tadic |  | 2014 |  | Y |  | Y |  | Y |  | Y |  | Y |  | N |  | Y |  | NS |  | NS |  | Y |  | Y |
| Tadic |  | 2018 |  | Y |  | Y |  | Y |  | Y |  | Y |  | N |  | Y |  | NS |  | NS |  | Y |  | Y |
| Van der Zwaan |  | 2010 |  | Y |  | Y |  | Y |  | Y |  | Y |  | N |  | Y |  | NS |  | NS |  | N |  | Y |
| Vitarelli |  | 2013 |  | Y |  | Y |  | Y |  | Y |  | Y |  | N |  | Y |  | NS |  | NS |  | Y |  | Y |
| Vitarelli |  | 2015 |  | Y |  | Y |  | Y |  | Y |  | Y |  | N |  | Y |  | Y |  | Y |  | Y |  | Y |
| Lakatos |  | 2020 |  | Y |  | Y |  | Y |  | Y |  | Y |  | N |  | Y |  | NS |  | NS |  | Y |  | Y |
| Addetia |  | 2018 |  | Y |  | Y |  | Y |  | Y |  | Y |  | N |  | Y |  | NS |  | NS |  | Y |  | Y |
| Aune |  | 2009 |  | Y |  | Y |  | Y |  | Y |  | Y |  | N |  | Y |  | N |  | N |  | N |  | Y |
| Gopal |  | 2007 |  | Y |  | Y |  | Y |  | Y |  | Y |  | N |  | Y |  | Y |  | Y |  | Y |  | Y |
| Kjaergaard |  | 2006 |  | Y |  | Y |  | Y |  | Y |  | Y |  | N |  | Y |  | NS |  | NS |  | N |  | Y |
| McGhie |  | 2016 |  | Y |  | Y |  | Y |  | Y |  | Y |  | N |  | Y |  | NS |  | NS |  | Y |  | Y |
| Tamborini |  | 2010 |  | Y |  | Y |  | Y |  | Y |  | Y |  | N |  | Y |  | NS |  | NS |  | Y |  | Y |
| Van Grootel |  | 2017 |  | Y |  | Y |  | Y |  | Y |  | Y |  | N |  | Y |  | NS |  | NS |  | Y |  | Y |
| D’Andrea |  | 2012 |  | Y |  | Y |  | Y |  | Y |  | Y |  | N |  | Y |  | Y |  | Y |  | Y |  | Y |
| D’Andrea |  | 2013 |  | Y |  | Y |  | Y |  | Y |  | Y |  | N |  | Y |  | Y |  | Y |  | Y |  | Y |
| Lai |  | 2015 |  | Y |  | Y |  | Y |  | Y |  | Y |  | N |  | Y |  | NS |  | NS |  | Y |  | Y |
| Lakatos |  | 2018 |  | Y |  | Y |  | Y |  | Y |  | Y |  | N |  | Y |  | NS |  | NS |  | Y |  | Y |
| Esposito |  | 2014 |  | Y |  | Y |  | Y |  | Y |  | Y |  | N |  | Y |  | NS |  | NS |  | N |  | Y |
| Vitarelli |  | 2015 |  | Y |  | Y |  | Y |  | Y |  | Y |  | N |  | Y |  | NS |  | NS |  | N |  | Y |
| Maffessani^a^ |  | 2013 |  | Y |  | Y |  | Y |  | Y |  | Y |  | N |  | Y |  | NS |  | NS |  | Y |  | Y |
| Zhang^a^ |  | 2016 |  | Y |  | Y |  | Y |  | Y |  | Y |  | N |  | Y |  | NS |  | NS |  | N |  | Y |
| Moceri^a^ |  | 2020 |  | Y |  | Y |  | Y |  | Y |  | Y |  | N |  | Y |  | NS |  | NS |  | N |  | Y |

*^a^Studies that met the inclusion criteria but were not included in meta-analyses (providing medians and interquartile range).*
