# Supplementary material for: Reference Values of Right Ventricular Volumes and Ejection Fraction by Three-Dimensional Echocardiography in Adults: A Systematic Review and Meta-Analysis
Source: Front Cardiovasc Med. 2021 Sep 23;8:709863. doi: 10.3389/fcvm.2021.709863 (PMC8495027; doi:10.3389/fcvm.2021.709863)
Supplement: Supplementary Figure 1 — The reference values for EDV and ESV by 3DE. [file Table_3.docx]

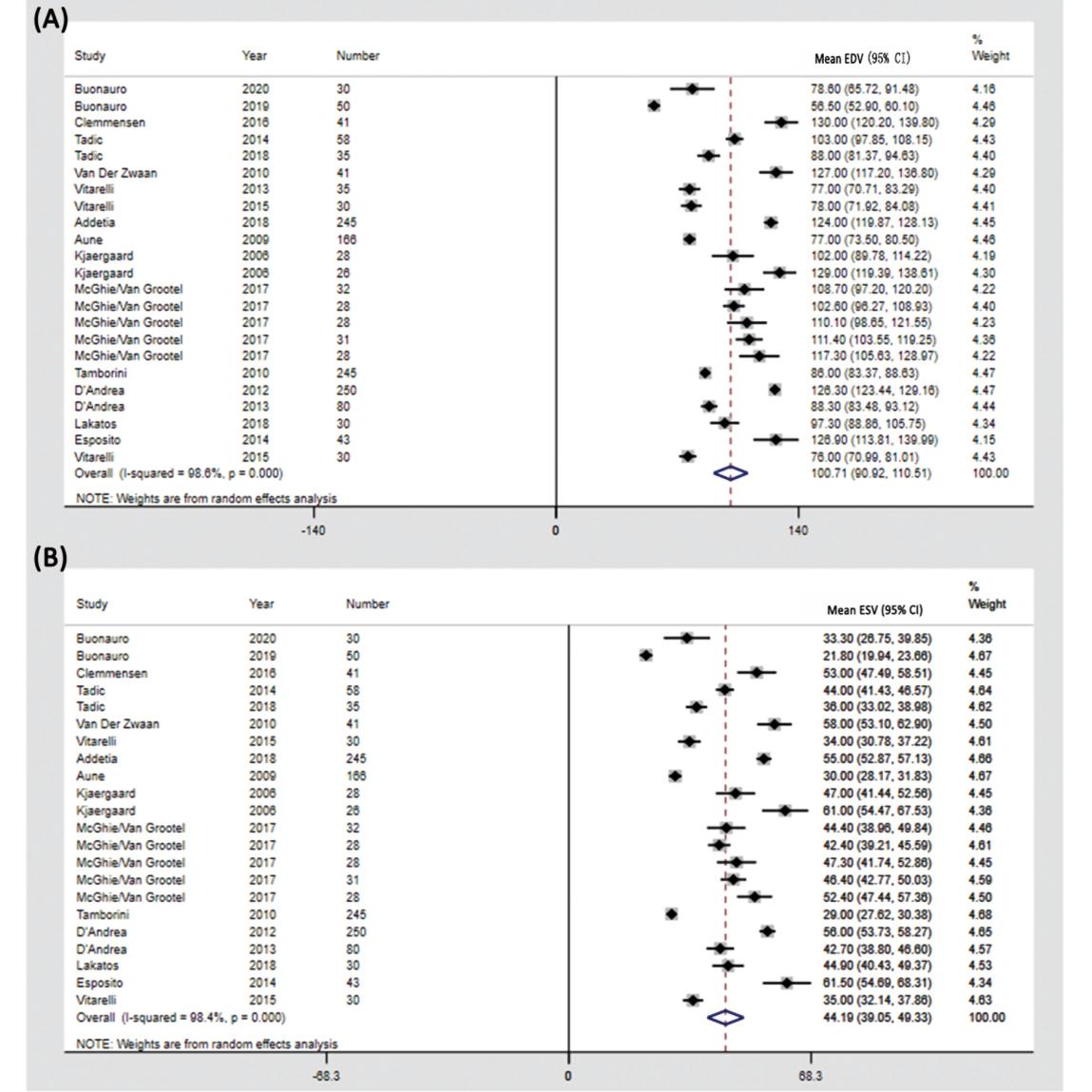


**Supplementary Figure 1.** The reference values for EDV and ESV by 3DE. (A) EDV, (B) ESV.

*The square represents the mean of the point effect estimate of each study. The square size indicates the weight of the study. The horizontal line extending from either side of the square represents the 95% CI. The diamond reflects the pooled overall consequence. 3DE, three-dimensional echocardiography; CI, confidence interval; EDV, end-diastolic volume; ESV, end-systolic volume.*
