# Supplementary material for: Reference Values of Right Ventricular Volumes and Ejection Fraction by Three-Dimensional Echocardiography in Adults: A Systematic Review and Meta-Analysis
Source: Front Cardiovasc Med. 2021 Sep 23;8:709863. doi: 10.3389/fcvm.2021.709863 (PMC8495027; doi:10.3389/fcvm.2021.709863)
Supplement: Supplementary Figure 3 — Funnel plots with ESV and ESVi estimates using the “trim and fill” method. [file Table_5.docx]

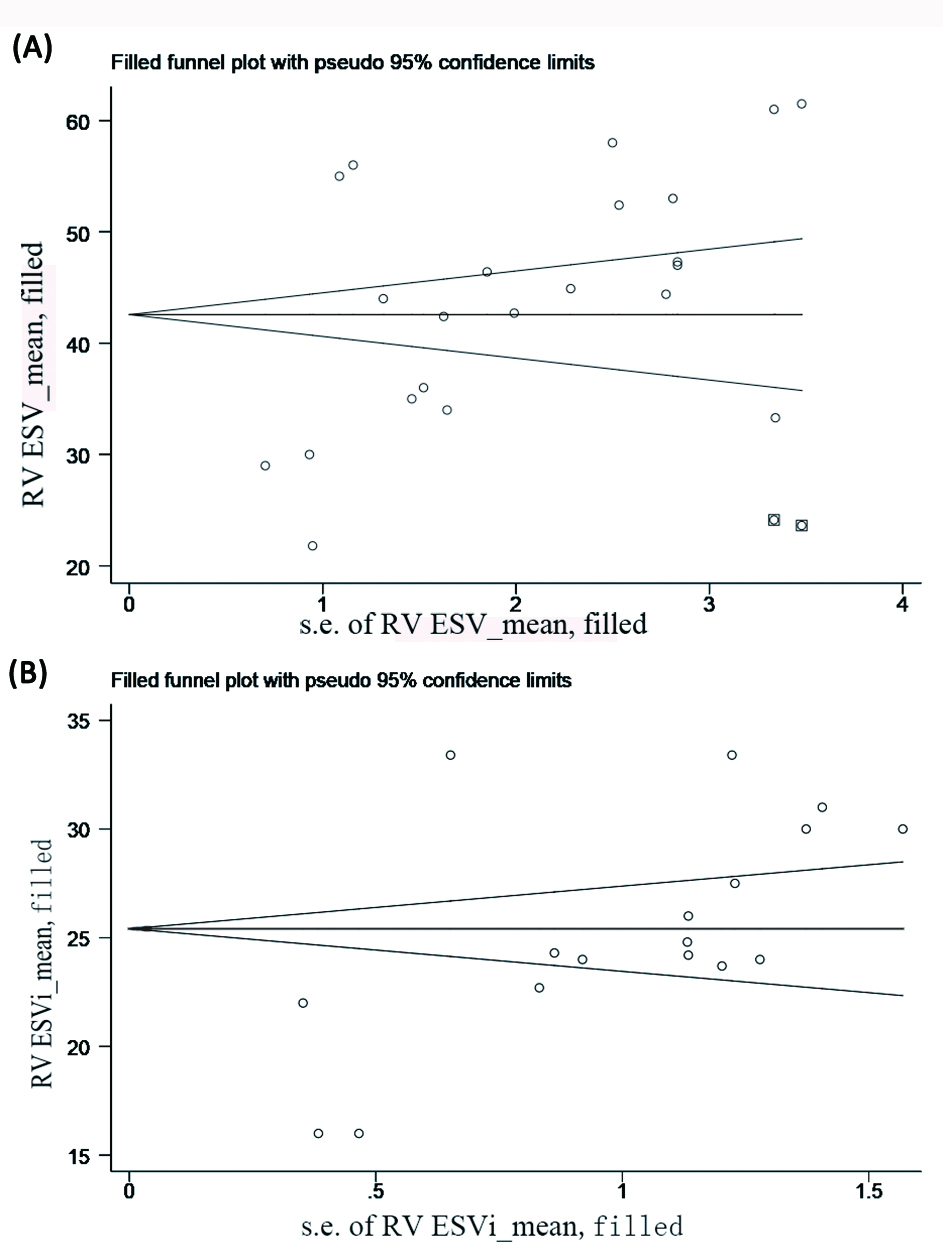


**Supplementary Figure 3**. Funnel plots with ESV and ESVi estimates using the “trim and fill” method. (A) RV ESV, (B) RV ESVi.

*The standard error of the effect estimate is plotted on the horizontal axis. The mean is plotted on the vertical axis. The circle presents each included study. The square presents the filled studies. The black lines indicate the pooled mean and 95% CI.*

*3DE, three-dimensional echocardiography; RV, right ventricular; ESV, end-systolic volume; ESVi, end-systolic volume indexed by body surface area.*
